# Efficacy of preoperative transcutaneous electrical acupoint stimulation in reducing postoperative delirium in older adults undergoing orthopedic surgery: a randomized controlled trial

**DOI:** 10.3389/fneur.2025.1668610

**Published:** 2025-10-27

**Authors:** Long Sun, Wen-Qing Ruan, Ying-Hua Zou, Pan Wei, He Li, Yong-Qiang Wang, Xing Li, Jian-Gang Song

**Affiliations:** Department of Anesthesiology, Shuguang Hospital Affiliated to Shanghai University of Traditional Chinese Medicine, Shanghai, China

**Keywords:** Hegu-Neiguan, older adults, orthopedic surgery, postoperative delirium, transcutaneous electrical acupoint stimulation, Zusanli-Taichong

## Abstract

**Background:**

Postoperative delirium (POD) is a common complication. Pain and sleep disturbances may contribute to the development of postoperative delirium, while transcutaneous electrical acupoint stimulation (TEAS) can alleviate these symptoms. This study investigated the effect of preoperative TEAS on POD prevention in older patients undergoing orthopedic surgery.

**Methods:**

A total of 608 patients were randomly assigned to either an intervention group (TEAS group) or a placebo group (control group). The TEAS group received stimulation at bilateral Hegu–Neiguan and Zusanli–Taichong acupoints for three consecutive days prior to surgery. POD was assessed twice daily using the Confusion Assessment Method from postoperative day 1 through day 5. Sleep disturbances were evaluated using the Athens Insomnia Scale (AIS), and pain was measured using the visual analog scale (VAS) on preoperative 3 days. Serum levels of interleukin-6 (IL-6), C-reactive protein (CRP), and S100β protein were quantified on postoperative days 1 and 3. In addition, cognitive function was evaluated on postoperative day 7 using the Montreal Cognitive Assessment (MoCA).

**Results:**

The incidence of POD was significantly lower in the TEAS group compared to the control group (6.08% vs. 17.57%, *p* < 0.001). Patients in the TEAS group also exhibited significantly lower serum levels of IL-6, CRP, and S100β on both postoperative days 1 and 3 (*p* < 0.05). Furthermore, significant improvements were observed in preoperative AIS scores and VAS scores for pain on days 1 and 2 prior to surgery in the TEAS group compared to the control group (*p* < 0.05). Besides, the MoCA score in the TEAS group was significantly higher than in the control group on postoperative day 7 (*p* < 0.05).

**Conclusion:**

Preoperative application of TEAS was associated with a reduced incidence of POD in older adults undergoing orthopedic surgery. The intervention may also contribute to improvements in preoperative sleep quality and pain reduction, as well as attenuation of postoperative inflammatory responses.

**Clinical trial registration:**

The trial was prospectively registered with the Chinese Clinical Trial Registry (Registration No. ChiCTR-INR-17012951; October 12, 2017).

## 1 Introduction

The growing number of older adults undergoing orthopedic procedures has brought increased attention to postoperative delirium (POD), a prevalent and clinically significant complication. The incidence of POD correlates positively with advancing age (1), highlighting its importance in this demographic. A comprehensive meta-analysis published in 2017 reported a cumulative incidence of POD as high as 24% among older adults undergoing orthopedic surgery (2). These data emphasize the urgent need for effective preventive strategies to improve postoperative outcomes and reduce healthcare burdens.

POD is associated with extended hospitalizations, heightened demands on healthcare personnel, and significant economic costs for families and society (3). Moreover, it contributes to elevated all-cause mortality, with reported risks ranging from 10 to 20% (4, 5). The long-term implications are equally concerning; delirium during hospitalization has been linked to increased post-discharge mortality and a markedly higher risk—up to tenfold—of subsequent dementia (6). Retrospective analyses of patients undergoing hip and knee arthroplasty have demonstrated a significantly greater likelihood of Alzheimer’s disease diagnosis in individuals with a prior history of delirium compared to those without such a history (7).

Established risk factors for POD include advanced age, diminished cognitive reserve, neuroinflammation associated with surgical or anesthetic trauma, acute pain, and preoperative sleep disturbances (8–11). Among these, pain and sleep disturbances represent modifiable factors that can be targeted to enhance perioperative brain health and reduce the risk of POD (12). Comprehensive perioperative management of these elements is essential for optimizing surgical outcomes and safeguarding patient well-being.

A substantial proportion of patients undergoing orthopedic procedures report significant preoperative pain; for example, up to 77% of patients scheduled for total knee arthroplasty experience notable discomfort (13). Sleep disturbances are also highly prevalent, with rates of 37% reported in patients with lumbar spinal stenosis and up to 73% in patients undergoing total hip arthroplasty (14, 15). Transcutaneous electrical acupoint stimulation (TEAS), a non-invasive method of electrical stimulation delivered through surface electrodes to specific acupoints, has demonstrated potential in addressing these perioperative challenges (16, 17). TEAS offers advantages over traditional acupuncture, including non-invasiveness, improved patient acceptability, and ease of clinical application. Preclinical and clinical studies have shown that TEAS is comparably effective to electroacupuncture in managing pain, modulating immune function, and treating substance use disorders (18–20).

Based on these observations, it was hypothesized that preoperative TEAS could reduce the incidence of POD by alleviating pain, improving sleep quality, and attenuating postoperative inflammatory responses. To evaluate this hypothesis, a randomized controlled trial was conducted among patients aged 65 years and older scheduled for orthopedic surgery. Participants received preoperative TEAS targeting specific acupoints (Zusanli ST36, Taichong LR3, Hegu LI4, and Neiguan PC6). The primary objective was to assess the efficacy of TEAS in the prevention of POD in this patient population.

## 2 Methods

This study was designed as a prospective, randomized, parallel-controlled clinical trial conducted in accordance with ethical principles outlined in the Declaration of Helsinki and relevant regulatory guidelines. Ethical approval was granted by the Ethics Committee of Shuguang Hospital, affiliated with Shanghai University of Traditional Chinese Medicine (Approval No. 2017–553–36-01; November 23, 2017). The trial was prospectively registered with the Chinese Clinical Trial Registry (Registration No. ChiCTR-INR-17012951; October 12, 2017).

Written informed consent was obtained from all participants prior to enrollment. Each participant was informed of the study’s purpose, procedures, potential risks, and their right to withdraw at any time. The ethical rigor and proper consent procedures employed in this study contribute to the reliability and generalizability of its findings.

### 2.1 Participants

Patients scheduled for orthopedic surgery were recruited based on predefined inclusion and exclusion criteria to ensure a clinically relevant and homogeneous study population. Inclusion criteria were: age > 65 years, either sex, scheduled for elective orthopedic surgery, American Society of Anesthesiologists (ASA) physical status classification I–III, and a body mass index (BMI) > 18 kg/m^2^ and ≤ 30 kg/m^2^. All participants were required to provide written informed consent prior to enrollment.

Exclusion criteria were: surgical scars or incisions at TEAS target acupoint sites (Zusanli ST36, Taichong LR3, Hegu LI4, or Neiguan PC6); local skin infections at these sites; emergency trauma surgery; participation in another clinical trial within four weeks before recruitment; use of a cardiac pacemaker; history of chronic pain persisting beyond normal tissue healing (typically >12 weeks); opioid addiction or dependence prior to surgery; cognitive impairment was screened using the Mini-Cognitive Assessment (Mini-Cog) instrument. A score of 0 was used as the exclusion criterion, indicating a high probability of dementia, as this tool is less influenced by educational level compared to other instruments (21); history of excessive alcohol use; or refusal to participate.

These criteria were designed to enhance the internal validity of the trial and ensure that the findings would be generalizable to a representative population of older adults undergoing orthopedic procedures.

### 2.2 Interventions

Participants were randomly allocated to either the intervention group (TEAS group) or the pseudo-TEAS placebo group (control group). Prior to surgery, a qualified nurse anesthetist identified the target acupoints: bilateral Zusanli (ST36)–Taichong (LR3) and Hegu (LI4)–Neiguan (PC6), totaling four pairs per participant.

In the intervention group, transcutaneous electrical stimulation was delivered using a low-frequency pulse generator (G6805-2; Shanghai Medical Instrument High-Tech Company). The device applied a dispersed-dense wave at 2/100 Hz, with stimulation intensity individualized to the maximum tolerable level for each patient. TEAS was administered twice daily for 30 min over three consecutive days prior to surgery. An additional 30-min session was administered on the day of surgery, commencing 30 min before induction of anesthesia.

In the control group, participants received sham stimulation using a modified electroacupuncture device that was visually indistinguishable from the active unit but delivered no electrical current. The pseudo-stimulation protocol matched the intervention schedule, with 30-min sessions administered twice daily for three days preoperatively and once more 30 min prior to anesthesia induction.

All interventions, including both active and sham stimulation, were conducted by the same trained nurse anesthetist to ensure procedural consistency. The orthopedic procedures and anesthetic management were performed by a consistent surgical and anesthesia team to maintain methodological rigor.

### 2.3 Anesthesia protocol

Upon entry to the operating room, standard physiological monitoring was initiated and maintained throughout the perioperative period for all participants. Parameters included electrocardiogram, pulse oxygen saturation, respiratory rate, end-tidal carbon dioxide, invasive arterial blood pressure, and bispectral index (BIS).

Anesthesia induction was performed using 0.3–0.5 μg/kg sufentanil, target-controlled infusion (TCI) of propofol at 3.0–6.0 μg/mL, and 0.9–1.2 mg/kg rocuronium bromide. Anesthesia maintenance included propofol via TCI at 2.5–4.5 μg/mL and remifentanil at 1.5–3.5 ng/mL. Anesthetic depth was adjusted to maintain a BIS range of 40–60. Hemodynamic stability was preserved by limiting intraoperative blood pressure fluctuations to within 20% of baseline, using volume resuscitation or cardiovascular agents as needed. Ten minutes before the end of surgery, 0.15 μg/kg sufentanil and 3 mg granisetron were administered to facilitate postoperative analgesia and mitigate postoperative nausea and vomiting.

In addition to general anesthesia, regional techniques were utilized based on surgical site and individual patient factors. For lower limb surgeries, spinal anesthesia was administered at the L2–L3 or L3–L4 interspace using 15–20 mg of 0.5% ropivacaine. In patients contraindicated for spinal anesthesia, general anesthesia with a laryngeal mask airway was combined with an ultrasound-guided fascia iliaca block (30 mL of 0.33% ropivacaine). For upper limb procedures, general anesthesia was similarly combined with an ultrasound-guided brachial plexus block (20 mL of 0.33% ropivacaine).

Postoperative analgesia was provided via a patient-controlled intravenous analgesia (PCIA) pump programmed with a continuous infusion of 1.5 μg/kg sufentanil and 6 mg granisetron at 2 mL/h. The bolus dose was set at 0.5 mL with a lockout interval of 15 min. Pain was assessed using the visual analog scale (VAS) during the first 24 h postoperatively. If the VAS score was ≥ 4 or additional analgesia was requested, 50 mg of intramuscular pethidine was administered as rescue therapy. This multimodal analgesic approach was designed to ensure effective pain control and support postoperative recovery.

### 2.4 Endpoints

The primary endpoint of this study was the incidence of POD within the first five days following surgery. POD was assessed by a nurse anesthetist blinded to group allocation, using the Confusion Assessment Method (CAM) or the CAM for the Intensive Care Unit (CAM-ICU) for intubated patients. Evaluations were conducted twice daily—once in the morning and once in the afternoon or evening—with a minimum interval of six hours between assessments, from postoperative day 1 through day 5.

Secondary endpoints included postoperative cognitive function, sleep quality, pain intensity, and serum biomarkers related to inflammation and brain injury. Early postoperative cognitive function was evaluated on postoperative day 7 using the Montreal Cognitive Assessment (MoCA). Sleep disturbances were assessed preoperatively on days −3, −2, and −1 using the Athens Insomnia Scale (AIS). Pain intensity was measured on the same preoperative days using the VAS.

Venous blood samples were collected preoperatively and on postoperative days 1 and 3. These samples were analyzed by the Clinical Laboratory of Shuguang Hospital to quantify levels of interleukin-6 (IL-6), C-reactive protein (CRP), and S100β protein, a biomarker of brain injury.

The comprehensive assessment of these primary and secondary outcomes was designed to elucidate the incidence and contributing factors of POD, as well as to explore the relationships among perioperative pain, sleep disturbances, neuroinflammation, and cognitive outcomes. This integrated approach aimed to inform strategies for improving perioperative care and clinical outcomes.

### 2.5 Sample size calculation

The sample size was determined based on the primary endpoint: the incidence of POD within the first five days after surgery. Participants were randomly allocated to either the TEAS group or the placebo (control) group. A total sample of 608 patients, with 304 in each group, was calculated to provide 80% power to detect a statistically significant difference at a two-sided alpha level of 0.05.

The calculation assumed an expected POD incidence of 24% in the control group and a 15% absolute reduction in the TEAS group, based on previously reported rates and anticipated treatment effect (2). Considering the possibility of potential loss to follow-up, we have factored in an additional 11% to the initially calculated sample size. Therefore, the final target enrollment for this study was set at 676 patients.

### 2.6 Randomization and blinding

Following informed consent, participants were randomized in a 1:1 ratio to either the TEAS group or the control group. Randomization was conducted using R software or equivalent randomization tools. Group assignments were concealed in sequentially numbered, sealed, opaque envelopes, which were placed in participants’ charts prior to surgery to maintain allocation concealment.

Given the perceptibility of electrical stimulation, a comprehensive blinding strategy was employed. All members of the clinical care team, including surgeons, anesthesiologists, and nurses, were blinded to treatment allocation. A designated nurse anesthetist, unblinded to group assignment, administered either the TEAS or pseudo-stimulation. In the event of intraoperative emergencies, the nurse anesthetist was permitted to unblind the treatment allocation if necessary to ensure patient safety.

Postoperative assessments were conducted by research personnel who remained blinded to group assignment. The study statistician was also blinded to group allocation for the duration of data analysis. All patient data, including group assignments, were stored in a password-protected spreadsheet on a secure research computer to maintain confidentiality and data integrity.

Participants were permitted to request disclosure of their treatment allocation after study completion. This blinding protocol, coupled with secure data management practices, was designed to minimize risk of bias and enhance the methodological rigor and credibility of the study’s findings.

### 2.7 Statistical methods for data analysis

The Kolmogorov–Smirnov test was used to assess the normality of distribution for continuous variables. For comparisons between groups, independent samples *t*-tests were applied to normally distributed variables with equal variances. If normality or homogeneity of variance was not met, the Mann–Whitney *U* test was used. Categorical variables were analyzed using the chi-squared (χ^2^) test or Fisher’s exact test, as appropriate.

For variables measured at multiple time points, such as VAS and AIS scores, repeated-measures analysis of variance (ANOVA) was conducted to assess within- and between-group differences over time. All statistical analyses were performed using SPSS software (version 22.0; SPSS Inc., Chicago, IL, USA). A two-tailed *p*-value of < 0.05 was considered statistically significant.

This statistical approach was designed to support robust and accurate interpretation of the data, enabling valid inference of treatment effects and intergroup differences.

## 3 Results

**Figure 1:**
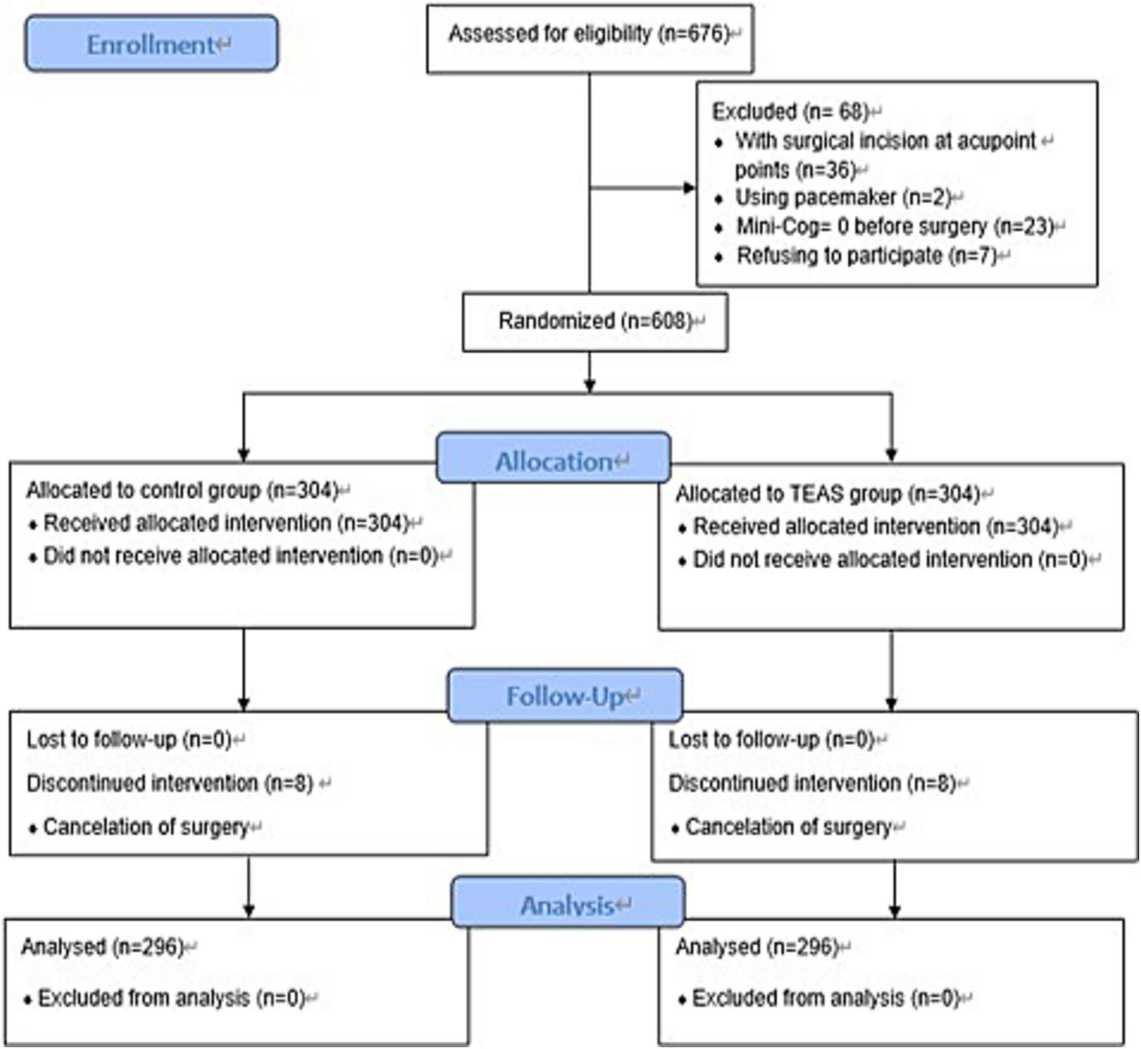
CONSORT flow diagram of patient enrollment, allocation, follow-up, and analysis.

Between December 10, 2017, and October 8, 2020, a total of 676 patients were screened for eligibility. Of these, 68 were excluded for the following reasons: 36 had surgical incisions at the acupoint sites, 2 had a pacemaker, 23 had a Mini-Cog score of 0, and 7 declined participation. The remaining 608 patients were randomized equally to the TEAS group and the control group (n = 304 per group). During the study period, 8 patients in each group withdrew due to surgical cancellations, resulting in 296 patients per group for final analysis (Figure 1).

Baseline demographic characteristics and perioperative data were comparable between the TEAS and control groups, indicating balanced distribution of key variables and minimizing potential confounding (Table 1).

**Table 1:** Demographic characteristics and surgical details.

Characteristic	Control group (*n* = 296)	TEAS group (*n* = 296)	*p* value
Age (years)	74.13 ± 4.58	73.80 ± 4.64	0.39
Female	180 (60.81)	175 (59.12)	0.68
BMI (kg/m^2^)	24.57 ± 2.71	24.21 ± 2.57	0.10
Level of education			0.66
Illiteracy	15 (5.07)	17 (5.74)	
Primary school	131 (44.26)	142 (47.97)	
Middle school	90 (30.41)	78 (26.35)	
High school	42 (14.19)	46 (15.54)	
University	18 (6.08)	13 (4.39)	
ASA classification			0.84
I	12 (4.05)	10 (3.38)	
II	235 (79.39)	233 (78.72)	
III	49 (16.55)	53 (17.91)	
Type of surgery			0.75
Traumatic	163 (55.07)	154 (52.03)	
Joint	74 (25)	80 (27.03)	
Spinal	59 (19.93)	62 (20.95)	
Anesthesia method			0.65
General	64 (21.62)	64 (21.62)	
Spinal	162 (54.73)	171 (57.77)	
General combined with nerve block	70 (23.65)	61 (20.61)	
Operation time (min)	145.32 ± 49.02	152.93 ± 52.01	0.07
Anesthesia time (min)	182.10 ± 51.66	188.15 ± 56.96	0.18

Values are presented as mean ± standard deviation, number (%).

ASA, American Society of Anesthesiologists; BMI, body mass index.

The incidence of POD was significantly lower in the TEAS group compared to the control group (6.08% vs. 17.57%, *p* < 0.001). Additionally, the TEAS group demonstrated significantly higher MoCA scores on postoperative day 7 (Table 2).

**Table 2:** Incidence of POD and postoperative MoCA score.

Characteristic	Control group (*n* = 296)	TEAS group (*n* = 296)	*P* value
Incidence of POD	52 (17.57%)	18 (6.08%)	< 0.001
Postoperative MoCA score	25.21 ± 1.43	27.28 ± 1.29	< 0.001

POD, postoperative delirium; MoCA, montreal cognitive assessment.

**Figure 2:**
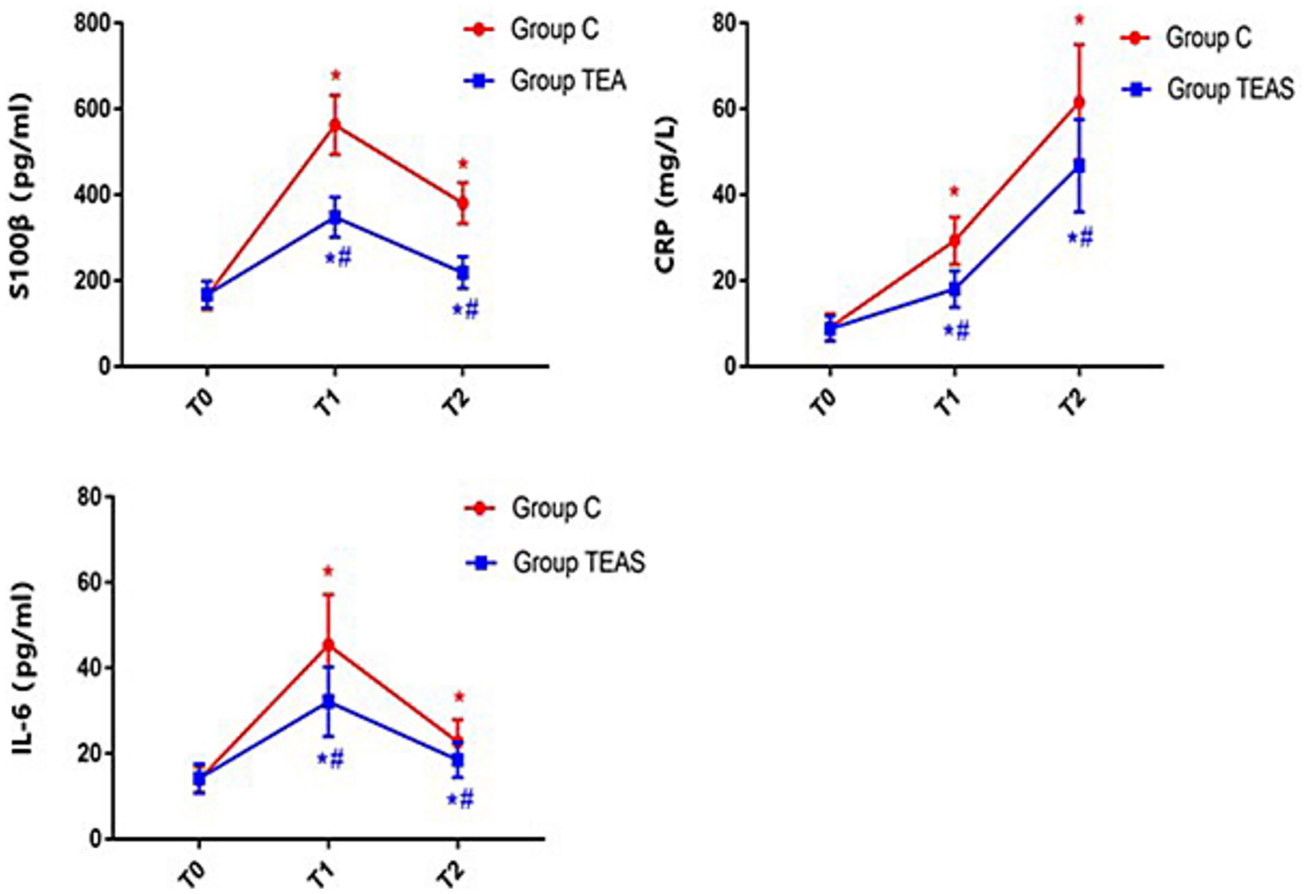
Serum concentrations of S100β, CRP, and IL-6 at defined perioperative time points. T0: pre-intervention (baseline); T1: postoperative day 1; T2: postoperative day 3. *p < 0.05 vs. baseline at T0; #*p* < 0.05 vs. control group.

Serum levels of S100β, CRP, and IL-6 increased significantly on postoperative days 1 and 3 in both groups compared to baseline (*p* < 0.05). However, the TEAS group exhibited significantly lower concentrations of these biomarkers on both postoperative days 1 and 3 compared to the control group (*p* < 0.05; Figure 2).

**Figure 3:**
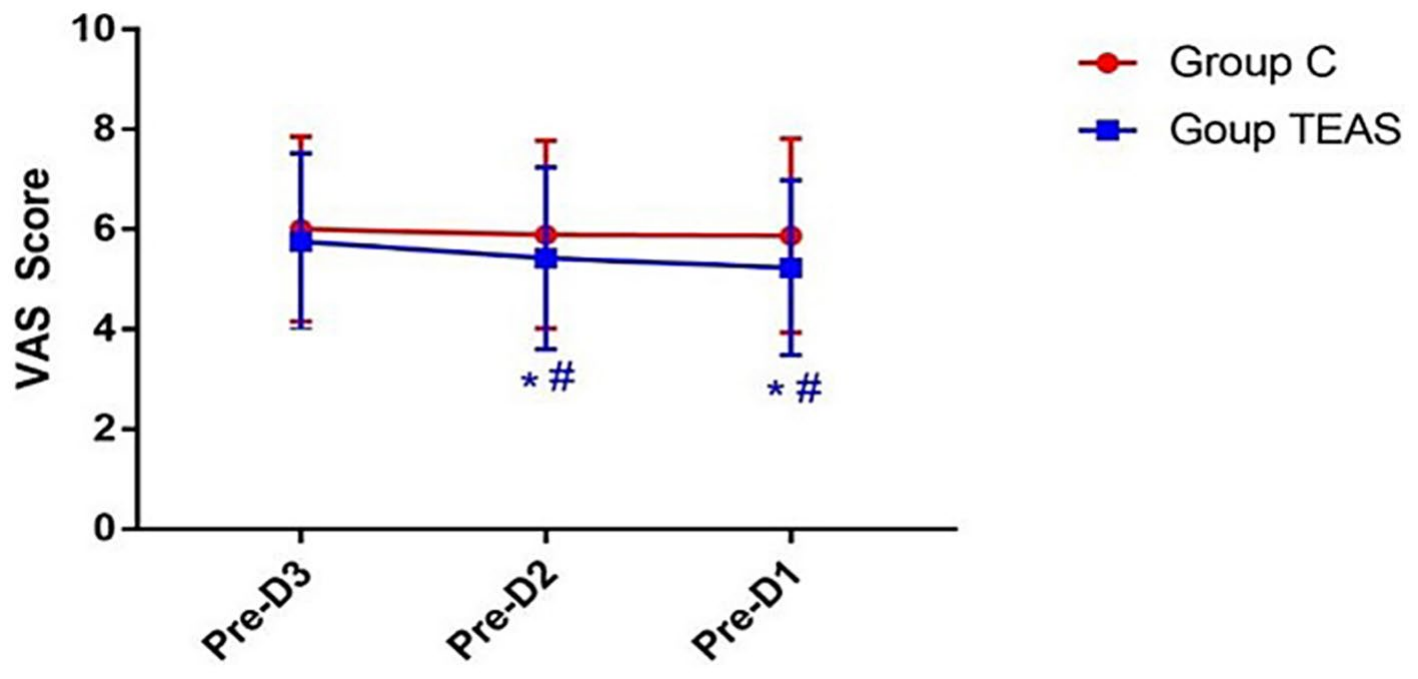
VAS pain scores measured on the three days prior to surgery. Pre-D3: 3 days before surgery; Pre-D2: 2 days before surgery; Pre-D1: 1 day before surgery. *p < 0.05 vs. Pre-D3; #*p* < 0.05 vs. control group.

The VAS scores remained unchanged in the control group across the three days preceding surgery. In contrast, the TEAS group showed a progressive decline in VAS scores, with significantly lower scores on preoperative days −2 and −1 compared to the control group (*p* < 0.05; Figure 3).

**Figure 4:**
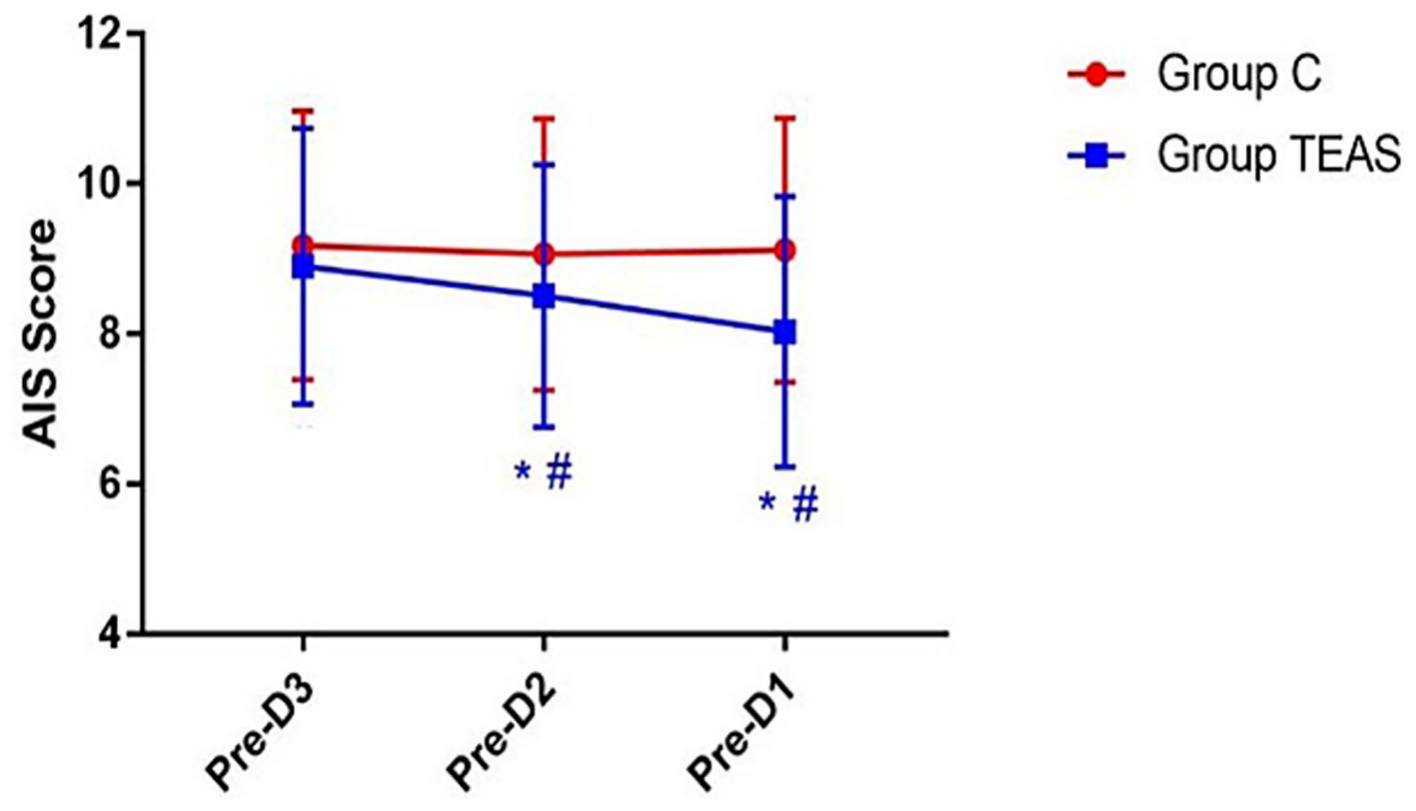
AIS scores recorded on the three days preceding surgery. Pre-D3: 3 days before surgery; Pre-D2: 2 days before surgery; Pre-D1: 1 day before surgery. *p < 0.05 vs. Pre-D3; #*p* < 0.05 vs. control group.

AIS scores in the TEAS group also showed a significant reduction over the three days prior to surgery. In the control group, AIS scores remained stable. On preoperative days −2 and −1, the TEAS group had significantly improved sleep scores relative to controls (*p* < 0.05; Figure 4).

The specific scoring data of the VAS and AIS scales was shown in the Supplementary Tables 1, 2.

## 4 Discussion

The present study provides compelling evidence supporting the efficacy of TEAS in reducing the incidence of POD and enhancing early postoperative cognitive function in older adults undergoing orthopedic surgery. Administration of TEAS over three consecutive days prior to surgery was associated with a significant reduction in POD incidence, suggesting its potential as a non-pharmacological strategy to improve perioperative neurocognitive outcomes in this demographic.

In addition to its impact on delirium prevention, TEAS demonstrated beneficial effects on several perioperative parameters, including preoperative pain, sleep quality, and postoperative inflammatory markers. Patients in the TEAS group exhibited reduced serum levels of S100β, CRP, and IL-6, indicating a suppression of systemic inflammation and neuroinflammatory response. These findings suggest a plausible mechanistic link between TEAS-mediated modulation of the inflammatory pathway and the observed improvements in cognitive outcomes.

These results are consistent with findings from prior studies. For example, a study involving 64 older adults with silent lacunar infarction undergoing spinal surgery reported a significant reduction in POD incidence in the TEAS group compared to controls (6.3% vs. 25%, *p* = 0.039) (22). Similarly, a single-center, prospective randomized trial demonstrated that a combined intervention of TEAS and auricular acupressure significantly reduced the incidence of POD in older adults undergoing major abdominal surgery (7.6% vs. 18.1%, *p* = 0.023) (23). Furthermore, a recent systematic review and meta-analysis concluded that TEAS is effective not only in reducing the incidence of POD but also in shortening its duration in older adults undergoing surgery (24).

Collectively, these findings contribute to a growing body of literature supporting the role of TEAS as a multifaceted intervention capable of addressing key perioperative risk factors for delirium, including pain, sleep disturbances, and inflammation. The consistency of findings across different surgical contexts reinforces the generalizability of TEAS as a preventive strategy for POD.

A distinctive feature of this study was the timing of the intervention. Unlike previous studies that initiated electroacupuncture 30 min before anesthesia induction, the current study implemented TEAS over three consecutive days prior to surgery. This decision was based on the clinical profile of the study population, in which a substantial proportion of patients presented with trauma-related conditions or were scheduled for joint replacement—scenarios often associated with significant preoperative pain. Approximately 77% of individuals undergoing total knee arthroplasty report moderate to severe pain preoperatively (13), and sleep disturbances are commonly linked to pain intensity (25). Addressing these modifiable risk factors in advance allowed for more comprehensive perioperative optimization, potentially reducing the neurocognitive burden associated with surgery.

Emerging evidence increasingly supports the relationship between preoperative sleep disturbances and POD. A prospective cohort study involving adults undergoing major non-cardiac surgery found that patients who developed POD experienced greater preoperative sleep fragmentation, including increased wakefulness after sleep onset and more frequent nocturnal awakenings (26). A meta-analysis encompassing 12 studies and 1,199 patients further confirmed that disrupted sleep is a significant predictor of POD onset (27). The results of our study showed that TEAS significantly improved the preoperative sleep scores. Actually, Existing evidence suggests that TEAS may exert its sleep improvement effect through: (1) Attenuating stress responses by suppressing hypothalamic–pituitary–adrenal axis hyperactivity and cortisol release; (2) Rebalancing sleep–wake neurotransmitters, including potentiation of GABAergic/5-HT signaling and inhibition of Orexin-A; (3) Enhancing melatonin synthesis to rectify circadian misalignment caused by hospitalization; (4) Anti-neuroinflammatory actions via dampening IL-6-mediated pathways (28).

Acupuncture analgesia, a key modality in traditional Chinese medicine, has demonstrated effectiveness in managing acute preoperative pain among older adults. A retrospective study involving 130 patients with femoral neck fractures found no significant differences in resting or activity-related pain scores, measured using the VAS, between those receiving acupuncture and those treated with femoral nerve blocks during the first two days after hospital admission (29). These findings suggest that acupuncture may provide analgesic benefits comparable to conventional nerve block techniques in this population.

The role of systemic inflammation in the pathogenesis of POD and postoperative cognitive dysfunction (POCD) is well established (30, 31). One prospective randomized controlled trial reported that inflammation within the central nervous system significantly contributes to POCD development (32). Preclinical and clinical evidence indicates that acupuncture pretreatment may attenuate systemic inflammatory responses. Notably, studies have shown that electroacupuncture applied to the Hegu (LI4) acupoint can inhibit endotoxin-induced inflammation, suppressing key pro-inflammatory mediators including interleukin-1β, tumor necrosis factor-*α* (TNF-α), and IL-6 (33, 34).

Supporting these findings, a recent animal study conducted by our team demonstrated that electroacupuncture reduced hippocampal inflammation and neuronal damage in a mouse model of POCD (35). In the present clinical study, TEAS—an intervention sharing mechanistic similarities with electroacupuncture—was associated with significantly lower serum levels of S100β, CRP, and IL-6 on postoperative days 1 and 3 compared to the control group. These results suggest that TEAS may mitigate neuroinflammation and reduce brain injury associated with surgical trauma.

Collectively, this body of evidence highlights the therapeutic potential of acupoint stimulation, including TEAS, in managing perioperative pain, dampening inflammatory responses, and protecting against surgery-related neurological injury. Incorporating TEAS as a non-pharmacological measure in perioperative care may contribute to reduced POD incidence, improved cognitive outcomes, and enhanced overall patient recovery.

## 5 Limitations

This study has several limitations. First, its single-center design may limit external validity and generalizability to broader populations or different clinical settings. Second, while the CAM was used to detect POD, the severity of delirium was not quantified using validated tools such as the Delirium Rating Scale-Revised-98 (DRS-R-98) (36). Third, in the control group, the absence of electrical stimulation, despite patient education, may have led to awareness of group assignment, particularly given the potential for interaction among participants treated in the same environment. Fourth, although outcome assessors were blinded, patients in the control group did not experience cutaneous stimulation, which may have undermined the integrity of patient blinding and influenced self-reported outcomes like pain and sleep quality. Fifth, the study did not capture the duration or severity of delirium episodes. Lastly, sleep assessment was limited to the subjective AIS, without objective measurements such as actigraphy, due to equipment and resource constraints. Additionally, the assessment of cognitive function was limited to a single timepoint (postoperative day 7) using the MoCA. While the MoCA is a validated screening tool for cognitive impairment, a formal diagnosis of POCD requires a battery of neuropsychological tests evaluating multiple domains and, crucially, a comparison to the patient’s own preoperative baseline performance to identify a clinically significant decline. Our study design did not include a preoperative cognitive assessment with the same instrument. Therefore, the higher MoCA scores in the TEAS group on day 7 should be interpreted as an indication of better early postoperative cognitive performance rather than a reduced incidence of POCD. Future studies designed specifically to investigate POCD should incorporate a comprehensive neuropsychological test battery administered both before and after surgery.

### 5.1 Future directions

Future research should aim to address these limitations through multi-center trials involving larger and more diverse cohorts. Delirium assessments should incorporate validated severity scoring systems (e.g., DRS-R-98, Memorial Delirium Assessment Scale) and, where feasible, neurophysiological monitoring such as electroencephalography. Methodologically, the development of inert stimulation devices capable of simulating tactile feedback would improve blinding integrity and reduce bias. Objective sleep assessments should complement subjective tools by including actigraphy and potential physiological biomarkers such as heart rate variability. Mechanistic investigations are also warranted, focusing on the optimal timing and duration of TEAS, its effects on neuroinflammatory pathways (e.g., IL-6, TNF-*α*, Tau protein), and changes in brain network function as measured by advanced imaging techniques like functional MRI. Future studies should also prioritize high-risk subgroups, such as individuals with baseline cognitive impairment or frailty, to further elucidate the therapeutic scope of TEAS in vulnerable populations.

## 6 Conclusion

TEAS significantly reduced the incidence of POD in older adults undergoing orthopedic surgery. This effect is likely mediated through improvements in preoperative sleep quality, effective pain control, and attenuation of systemic inflammatory responses. These findings support the utility of TEAS as a non-invasive, adjunctive intervention for enhancing perioperative care and improving neurocognitive outcomes in this high-risk surgical population.

## Data Availability

The original contributions presented in the study are included in the article/Supplementary material, further inquiries can be directed to the corresponding authors.
